# Chaga mushroom-induced oxalate nephropathy that clinically manifested as nephrotic syndrome: A case report

**DOI:** 10.1097/MD.0000000000028997

**Published:** 2022-03-11

**Authors:** Ohyun Kwon, Yaerim Kim, Jin Hyuk Paek, Woo Yeong Park, Seungyeup Han, Hyungchan Sin, Kyubok Jin

**Affiliations:** a Division of Nephrology, Department of Internal Medicine, Keimyung University School of Medicine, Keimyung University Dongsan Hospital, Daegu, Republic of Korea; b Keimyung University Kidney Institute, Daegu, Republic of Korea; c Department of Pathology, Keimyung University School of Medicine, Keimyung University Dongsan Hospital, Daegu, Republic of Korea.

**Keywords:** acute kidney injury, calcium oxalate, *Inonotus obliquus*, nephrotic syndrome

## Abstract

**Rationale:**

The Chaga mushroom (Hymenochaetaceae*, Inonotus obliquus*) is a fungus belonging to the Hymenochaetaceae family. It is parasitic on birch and other tree species. Chaga mushrooms are rich in various vitamins, minerals, and nutrients. Some people consider these mushrooms medicinal as they have been reported to suppress cancer progression through anti-inflammatory and antioxidant effects. However, recent studies have reported that excessive ingestion of Chaga mushrooms can cause acute oxalate nephropathy.

**Patient concerns:**

A 69-year-old man who ingested Chaga mushroom powder (10–15 g per day) and vitamin C (500 mg per day) for the past 3 months developed acute kidney injury (AKI) with the clinical manifestations of nephrotic syndrome (NS).

**Diagnosis:**

Pathological findings showed focal acute tubular injury and the deposition of calcium oxalate crystals in the tubules. Light microscopy showed interstitial fibrosis and tubular atrophy, and electron microscopy showed the effacement of the foot processes in podocytes. Based on these results, the diagnosis was acute oxalate nephropathy accompanied by minimal change disease (MCD).

**Interventions:**

The patient's kidney function did not improve with supportive care, such as hydration and blood pressure control. Thus, we recommended hemodialysis and the administration of a high dose of steroids (intravenous hydrocortisone 500 mg twice a day for 3 days and oral prednisolone at 1 mg/kg).

**Outcomes:**

The patient's kidney function recovered just 1 month after the start of treatment, and the MCD was completely remitted.

**Lessons:**

In cases of AKI with an unknown cause, it is important to closely observe the patient's medication history, and it is recommended to perform kidney biopsy. Furthermore, this study showed that active dialysis and high-dose steroid treatment can restore kidney function in patients with AKI caused by acute oxalate nephropathy with MCD.

## 1 Introduction

The Chaga mushroom is one of the most popular remedies in folk medicine because of its rich vitamins, nutrients, and anti-inflammatory^[1]^ and antioxidant effects.^[2]^ However, it has been reported that the ingestion of excessive Chaga mushroom can result in acute oxalate nephropathy caused by the deposition of calcium oxalate crystals in the renal tubules.^[3]^ This leads to acute kidney injury (AKI) and chronic renal tubular changes, such as interstitial fibrosis and tubular atrophy. Acute oxalate nephropathy is characterized by generalized edema, oliguria, confusion, and cardiac arrhythmias based on the severity of AKI.^[4]^ Nevertheless, there are few reports of acute oxalate nephropathy with a feature of nephrotic syndrome (NS).

Here, we report a case of acute oxalate nephropathy with NS in a male patient who ingested excessive Chaga mushroom.

## 2 Case report

A 69-year-old Korean man with generalized edema and oliguria was transferred to our medical center. He visited a local medical center for generalized edema and oliguria that have persisted for 3 days. He had benign prostate hypertrophy, but did not have hypertension or diabetes mellitus. During the patient's medical checkup 8 months ago, his serum creatinine level was 1.3 mg/dL; he had no family history of kidney diseases. He was prescribed finasteride (5 mg once a day) and terazocin (2 mg once a day) for the maintenance of benign prostate hypertrophy. He also took Chaga mushroom powder (Korea Ginseng Co.), which he understood to be effective in boosting immunity, at a dosage of 10 to 15 g per day for the past 3 months.

**Figure 1:**
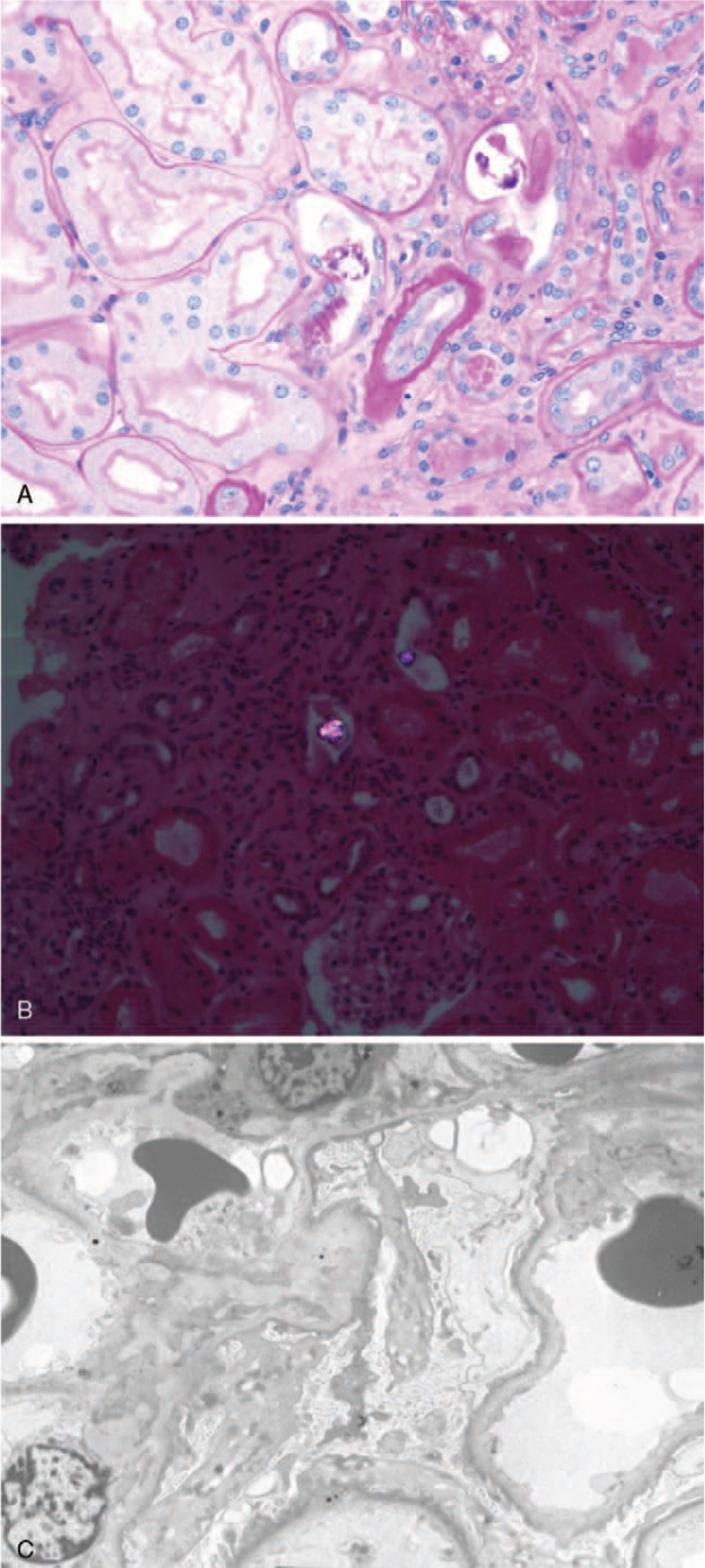
Renal biopsy of a patient with oxalate nephropathy secondary to ingestion of Chaga mushroom. (A) Hematoxylin and eosin staining viewed under light microscopy showed focal acute tubular injury, deposition of calcium oxalate crystals in tubules, interstitial fibrosis, and tubular atrophy. (B) The calcium oxalate crystals demonstrated birefringence under polarized light. (C) Electron microscopy showed that there was an effacement of foot processes in podocytes.

At our local medical center, the patient had a blood urea nitrogen (BUN) level of 104 mg/dL, serum creatinine of 6.24 mg/dL, and estimated glomerular filtration rate of 10 mL/min/1.73 m^2^. On admission, he had a height of 175.0 cm, body weight of 68.2 kg, and body mass index of 22.3 kg/m^2^. His blood pressure was 160/84 mm Hg, pulse rate was 80 beats/min, and body temperature was 36.5°C. Moderate pretibial edema was observed. There were no skin lesions on the whole body. A urine dipstick test revealed proteinuria (3+, 500 mg) and occult blood (3+). Urine sediment examination showed 6 to 10 red blood cells and 6 to 10 white blood cells per high-power field with no casts. He had a urinary protein-to-creatinine ratio of 5.78 g/g. The patient also had the following test results: total cholesterol of 286 mg/dL, low-density lipoprotein–cholesterol of 197 mg/dL, hemoglobin of 12.2 g/dL, hematocrit of 35.6%, white blood cell count of 6,670/μL, platelet count of 15.5 × 10^4^/μL, serum total protein of 6.4 g/dL, serum albumin of 2.9 g/dL, BUN of 105 mg/dL, serum creatinine of 6.94 mg/dL, cystatin C level of 4.77 mg/L, estimated glomerular filtration rate of 7.9 mL/min/1.73 m^2^, uric acid of 13.2 mg/dL, and fasting blood sugar level of 103 mg/dL. The serum electrolyte levels were as follows: sodium 135 mmol/L, potassium 5.3 mmol/L, chloride 101 mmol/L, calcium 8.1 mg/dL, and inorganic phosphate 5.3 mg/dL. Liver function tests revealed the following findings: aspartate transaminase of 19 U/L, alanine transaminase of 10 U/L, and alkaline phosphatase of 80 U/L. The results of immunological examination were within the normal range (serum levels of immunoglobulin[Ig] G, 1482 mg/dL; IgA, 331 mg/dL; IgM, 71 mg/dL; C3, 92.0 mg/dL; C4, 29.4 mg/dL). Electrocardiography revealed no arrhythmia and echocardiography revealed normal findings. Chest X-ray of the posteroanterior view showed no cardiomegaly or pulmonary edema. Kidney ultrasonography revealed diffusely increased echogenicity, and the kidney size was within the normal range. Despite hydration and medical treatment, the oliguria and renal function of the patient did not improve. Due to generalized edema and uremic symptoms, emergent hemodialysis via a permanent catheter was started and renal biopsy was performed on the second day of hospitalization to evaluate the deteriorated kidney function. Pathological findings revealed focal acute tubular injury, deposition of calcium oxalate crystals in tubules, interstitial fibrosis, and tubular atrophy on examination by light microscopy (Fig. 1A and B). Immunofluorescence showed that IgG, IgA, IgM, C3, C4, C1q, and light chains in the glomerulus were not expressed. Electron microscopy revealed effacement of the foot processes in podocytes (Fig. 1C). The pathologic findings indicated that the patient was in the recovery phase of acute tubular injury with oxalate crystal deposits and may have minimal change disease (MCD).

The patient had clinical manifestations of NS and AKI. Based on the pathologic findings and the patient's herbal medication history, we considered 2 diagnoses: oxalate nephropathy and MCD. Therefore, a high-dose of intravenous steroids (250 mg of hydrocortisone twice a day for 3 days) and oral prednisolone (1 mg/kg per day) were administered concurrently with hemodialysis. Renal function recovered (BUN and serum creatinine became 16 mg/dL and 1.00 mg/dL, respectively) 1 month after the beginning of treatment, so hemodialysis was stopped. NS also seemed to be fully remitted, as indicated by the urine protein creatinine ratio of 0.48 g/g.

## 3 Discussion

Although not clinically validated, AKI is often experienced as a side effect due to the intake of several medications, including herbal medications or healthy foods. These medications or foods generally emphasize anti-inflammatory and antioxidant effects and report overall effects rather than their mechanism of action on specific diseases. However, their efficacy and safety have not been proven by large-scale clinical trials or scientific methods, thus often leading to harmful side effects. Chaga mushroom is one such herbal medication that has been reported in several studies to have anti-inflammatory and antioxidant effects.^[1,2]^ However, ingesting high doses of Chaga mushroom can cause acute oxalate nephropathy, which can lead to acute kidney damage.^[5]^ The prognosis of AKI differs depending on the cause. In the case of Chaga mushroom-induced AKI, patients have been reported to improve only with conservative treatment or, in severe cases, have been required to undergo dialysis treatment for maintenance but without a significant improvement in the condition.^[6]^ In our case, AKI and general edema worsened enough to require dialysis, and clinical symptoms similar to those of NS developed. Hemodialysis was performed until the urine volume was maintained and edema improved. The kidney biopsy showed the presence of calcium oxalate crystals with focal acute tubular injury and effacement of the foot processes – results that are expected of MCD patients. Therefore, high-dose steroids were prescribed, and proteinuria and AKI dramatically improved. In previous studies, the treatment of calcium oxalate deposition has not been well established.^[5]^ Furthermore, oxalate crystals cannot be eliminated by hemodialysis. It can be controversial whether MCD was treated by steroids or just by stopping mushroom ingestion and waiting in this study.^[7]^ However, MCD can be managed with steroid therapy, which usually results in a good response. Considering that the biopsy showed MCD and the NS state improved rapidly after high-dose steroid treatment, it is difficult to say that the clinical course of the patient improved dramatically just by stopping the drug. Therefore, we opted for conservative management with hemodialysis and high-dose steroid therapy for the patient, and his kidney function recovered completely. It is not clear which treatment is effective in acute oxalate nephropathy with NS, but it may be advisable to try high-dose steroid treatment when the patient has clinical manifestations of NS.

This case report had some limitations. First, we could not determine the urinary oxalate concentration after 24 hours. Second, we did not perform an additional biopsy after treatment, so we could not confirm the kidney recovery based on histologic findings. However, the patient completely recovered from AKI and MCD based on clinical manifestations. Further research is needed to clarify the disease course of Chaga mushroom-induced oxalate nephropathy.

## 4 Conclusions

We report a case in which a patient with Chaga mushroom-induced oxalate nephropathy and NS recovered completely through high-dose steroid treatment and hemodialysis. In cases of AKI with an unknown cause, it is therefore important to closely examine the patient's medication history, and actively perform a kidney biopsy. It is also recommended to administer high-dose steroids to the patient.

## Author contributions

**Conceptualization:** Woo Yeong Park.

**Resources:** Yaerim Kim, Jin Hyuk Paek, Seungyeup Han, Hyungchan Shin.

**Supervision:** Kyubok Jin.

**Writing – original draft:** Ohyun Kwon.

**Writing – review & editing:** Woo Yeong Park, Kyubok Jin.
